# Patients with systemic lupus erythematosus have impaired strain and vascular function which is incremental to that caused by traditional risk factors: insights from Cardiovascular Magnetic Resonance

**DOI:** 10.1186/1532-429X-15-S1-E56

**Published:** 2013-01-30

**Authors:** Ntobeko A Ntusi, Jane M Francis, Paul M Matthews, Paul B Wordsworth, Stefan Neubauer, Theodoros Karamitsos

**Affiliations:** 1 Cardiovascular Medicine, University of Oxford, Oxford, UK; 2 GSK Clinical Imaging Centre, Imperial College, London, UK; 3 Bortnar Institute and Nuffield Department of Orthopaedics, Rheumatology and Musculoskeletal Sciences, University of Oxford, Oxford, UK

## Background

Systemic lupus erythematosus (SLE) is a systemic autoimmune disorder that commonly affects the heart. The impact of SLE on the heart is a 7 to 9 times greater incidence of cardiovascular disease (CVD) in SLE patients compared to healthy controls. Moreover, female patients with SLE between 35 and 44 years old have an incidence of myocardial infarction over 50 times greater than that observed in the Framingham cohort. The exact cause of this excess CVD burden in SLE is poorly understood, but is thought to be multi-factorial. Cardiovascular magnetic resonance (CMR) has the capacity of simultaneously assessing non-invasively cardiac function, altered vascular distensibility, myocardial strain and fibrosis. The purpose of this study was to assess cardiac and vascular function and myocardial strain in patients with SLE and to determine their relation to the presence of cardiovascular risk factors (CVRFs) and SLE disease duration.

## Methods

11 SLE patients with no CVRFs (11 female, mean age 37 ± 7), 19 SLE patients with CVRFs (18 female, mean age 47 ± 11), 39 normal controls (39 female, mean age 45 ± 12), and 11 controls with CVRFs (11 female, mean age 52 ± 9), underwent CMR at 1.5 Tesla. All patients with previously known CVD were excluded. CVRFs, disease activity index and duration of disease were recorded for each subject. Biventricular volumes and function, LGE, myocardial strain and vascular function were assessed by CMR. Aortic distensibility and pulse wave velocity (PWV) were measured in the ascending aorta, proximal descending aorta and distal descending aorta.

## Results

There were no differences in left ventricular (LV) volumes and LV ejection fraction between the 4 groups (Table 1). SLE patients with CVRFs showed the greatest reduction in mid short axis circumferential systolic strain, peak diastolic strain rate, and vascular indices. SLE patients without CVRFs showed a similar degree of vascular dysfunction and deformational abnormality as controls with CVRFs. Aortic distensibility (Rs= -0.59, p<0.001) and total pulse wave velocity (Rs= 0.29, p=0.01) correlated with SLE disease duration (Table 2).

**Table 1:** Demographic, clinical and CMR features in SLE, SLE with CVRFs, controls and controls with CVRFs.

	Normal controls N=39	Controls with CVRFs N=11	SLE N=11	SLE with CVRF N=19	P Value
Age (years)	44.6 ± 11.8	51.7 ± 8.9	37.0 ± 7.0	46.7 ± 10.7	0.02

Females (%)	38 (97.4)	11 (100)	11 (100)	18 (94.7)	0.77

BMI (kg/m^2^)	22.5 ± 2.3	27.5 ± 5.2	24.4 ± 2.5	30.5 ± 6.4	<0.001

LVEDV (ml) indexed to BSA	76.6 ± 12.1	79.3 ± 20.2	80.7 ± 16.1	70.3 ± 14.6	0.22

LVESV (ml) indexed to BSA	21.1 ± 5.4	19.8 ± 6.1	23.0 ± 10.5	20.3 ± 6.4	0.66

LVEF	72.5 ± 4.1	75.1 ± 5.3	72.6 ± 7.3	71.7 ± 3.7	0.27

LA size	2.7 ± 0.5	2.9 ± 0.5	3.0 ± 0.4	3.3 ± 0.6	<0.001

BMI, Body mass index; LVEDV, Left ventricular end diastolic volume; BSA, Body surface area; LVESV, Left ventricular end systolic volume; LVEF, Left ventricular ejection fraction; LA, left atrium.

**Table 2:** Systolic circumferential strain, aortic distensibility and pulse wave velocity in SLE, SLE with CVRFs, controls and controls with CVRFs.

	Normal controls N=39	Controls with CVRFs N=11	SLE N=11	SLE with CVRF N=19	P Value
Mid SA systolic circumferential strain	-19.4 ± 1.1	-18.4 ± 1.4	-16.7 ± 1.2	16.3 ± 1.0	<0.001

Peak diastolic strain rate	144.5 ± 14.5	126.9 ± 20.7	99.6 ± 23.2	89.4 ± 17.3	<0.001

Aortic distensibility (10^-3^mmHg^-1^) Ascending aorta Proximal descending aorta Distal descending aorta	3.6 ± 2.0 4.1 ± 1.5 6.2 ± 2.5	3.1 ± 1.9 3.4 ± 1.7 4.7 ± 1.8	3.2 ± 1.1 3.8 ± 1.0 5.0 ± 1.2	2.4 ± 1.2 2.8 ± 1.0 3.6 ± 1.2	0.11 0.006 <0.001

Pulse wave velocity (m/s) Aortic arch PWV Descending aorta PWV Total PWV	4.2 ± 2.0 3.7 ± 1.5 4.3 ± 1.4	5.5 ± 1.9 5.9 ± 1.6 5.4 ± 2.4	6.0 ± 1.2 6.6 ± 1.6 6.3 ± 1.3	7.5 ± 2.1 8.1 ±1.7 8.5 ± 1.9	<0.001 <0.001 <0.001

SA, Short axis; PWV, pulse wave velocity.

## Conclusions

Evidence of impaired circumferential systolic strain and vascular function in SLE is demonstrated on CMR assessment, which is independent and incremental to that due to traditional CVRFs.

## Funding

NN is funded by the Discovery Foundation and the Nuffield Trust. SN acknowledges support from the British Heart Foundation Centre of Research Excellence, Oxford. The research was funded by an investigator-led grant from GSK.

